# Evaluation of the anti-hyperglycemic effect and safety of microorganism 1-deoxynojirimycin

**DOI:** 10.1371/journal.pone.0199057

**Published:** 2018-06-13

**Authors:** Soo Takasu, Isabella Supardi Parida, Shinji Onose, Junya Ito, Ryoichi Ikeda, Kenji Yamagishi, Oki Higuchi, Fukuyo Tanaka, Toshiyuki Kimura, Teruo Miyazawa, Kiyotaka Nakagawa

**Affiliations:** 1 Food and Biodynamic Chemistry Laboratory, Graduate School of Agricultural Science, Tohoku University, Sendai, Japan; 2 Food Research Laboratory, Asahimatsu Foods Co., Ltd., Iida, Nagano, Japan; 3 Food Research Institute (NFRI), National Agriculture and Food Research Organization (NARO), Tsukuba, Ibaraki, Japan; 4 Biodynamic Plant Institute Co., Ltd., Sapporo, Hokkaido, Japan; 5 Central Region Agricultural Research Center, National Agriculture and Food Research Organization (NARO), Tsukuba, Ibaraki, Japan; 6 New Industry Creation Hatchery Center (NICHe), Tohoku University, Sendai, Japan; The University of Tokyo, JAPAN

## Abstract

1-Deoxynojirimycin (DNJ) is a potent α-glucosidase inhibitor and thus beneficial for prevention of diabetes. While we have succeeded in obtaining the culture supernatant extract (CSE) rich in DNJ from microorganism source, information regarding its anti-hyperglycemic effect and safety were still limited. Therefore, this study was aimed to evaluate the anti-hyperglycemic effect and safety of microorganism DNJ. Oral sucrose tolerance test was performed, and the result showed that CSE was able to significantly suppress the blood glucose elevation and suggested DNJ as the main active compound. To determine its safety, the absorption and excretion of microorganism DNJ were evaluated using ^15^N labeling method. Our findings investigated the recovery rate of ^15^N from DNJ reached 80% up to 48 hours after oral administration, suggesting its rapid excretion, suggesting the safety of DNJ. This study verified the functional properties and safety of DNJ from microorganisms, suggesting its potential use for functional purpose.

## Introduction

**Fig 1:**
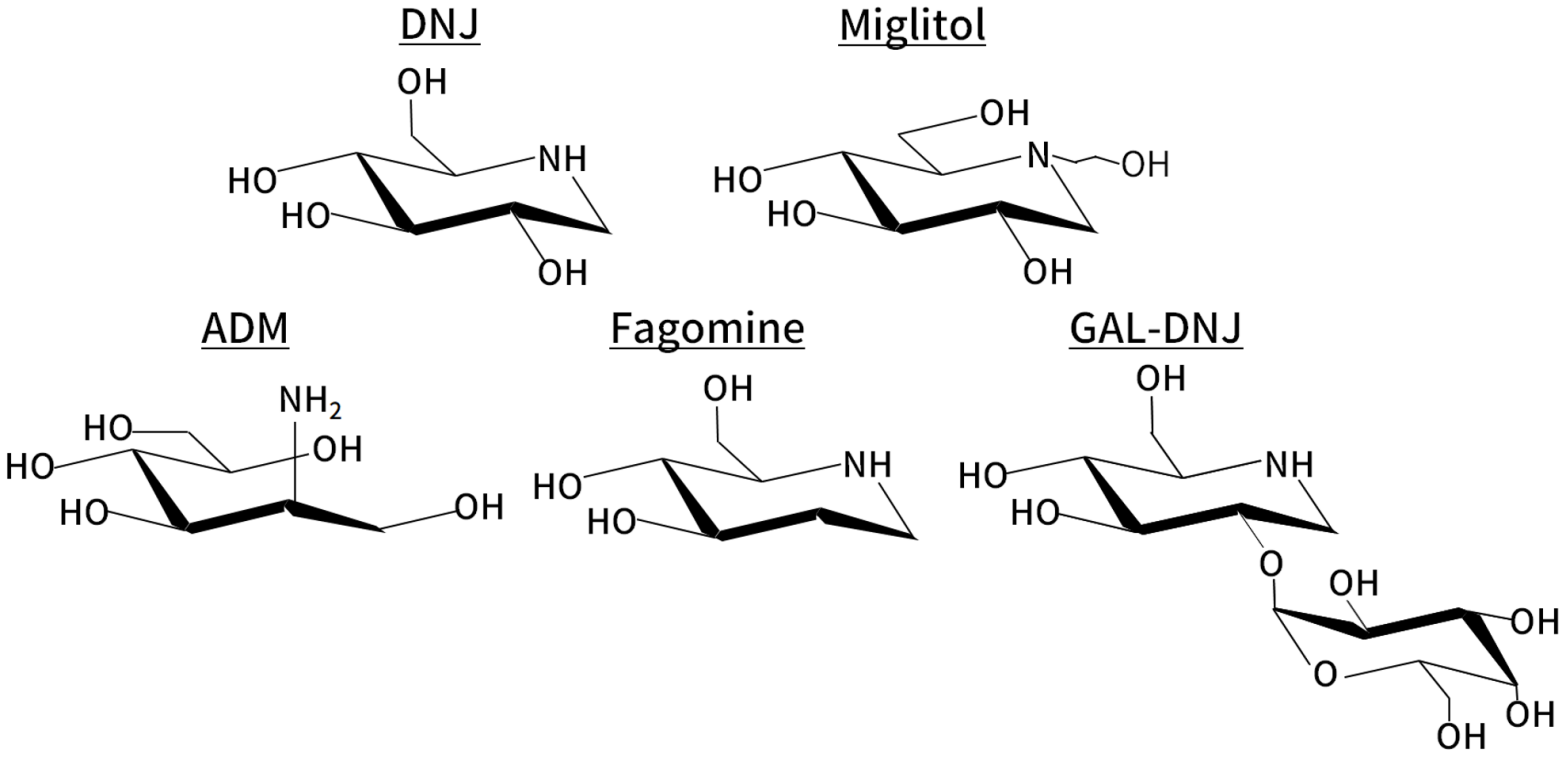
Chemical structures of aza-sugars. Chemical structures of 1-Deoxynojirimycin (DNJ), miglitol, 2-amino-2-deoxy-D-mannitol (ADM), fagomine, and 2-O-α-D-galactopyranosyl-deoxynojirimycin (GAL-DNJ).

Mulberry leaves have long been used in traditional treatment for diabetes. In recent years, it has been revealed that mulberry leaves extract (MLE) suppresses postprandial hyperglycemia via α-glucosidase inhibition [1]. The active component of MLE was identified as 1-deoxynojirimycin (DNJ) [2], a typical naturally occurring aza-sugar with an imino group (-NH-) substituting for the oxygen atom in the pyranose ring (Fig 1). Since DNJ has a potent α-glucosidase inhibitory activity [3], it has been used as a lead compound in the development of anti-diabetic drug such as miglitol. Besides its anti-hyperglycemic effect, MLE is also expected to improve lipid metabolism [4]. Considering its health benefits, MLE has been utilized as various functional foods. However, only low amount of DNJ can be obtained from mulberry leaves (approximately 0.1% w/w) [5], leading to high production cost. Moreover, the quantity of mulberry leaves has been declining, as the result of considerable decline in Japanese silk production.

The production of DNJ by *Bacillus* sp. [6] and *Streptomyces* sp. [7] have been reported. A gene cluster that initiates the aza-sugar biosynthesis in *Bacillus amyloliquefaciens* was also identified [8]. Recently, we succeeded in increasing the production of DNJ by optimizing the culture conditions (e.g. carbon source) of *B*. *amyloliquefaciens* [9]. After the culture medium was subjected to extraction, culture supernatant extract (CSE) rich in DNJ could be obtained. Hence, the application of DNJ-producing microorganisms is expected to decrease production costs, while at the same time increase the supply of DNJ. However, unlike MLE [10,11], the anti-hyperglycemic effect and safety of DNJ from microorganisms have not been well studied.

Therefore, in this study, we evaluated the anti-hyperglycemic effect of CSE in comparison to MLE, miglitol and DNJ using oral sucrose tolerance test (OSTT) in rats. To determine the safety of DNJ, we observed its absorption and excretion using ^15^N-labeled DNJ from microorganism source. The ^15^N-labeled DNJ was orally administered to rats and its absorption and excretion rate were measured based on the nitrogen isotope content in urine and feces.

## Materials and methods

### Materials

DNJ standard was purchased from Sigma-Aldrich Japan (Tokyo, Japan). Miglitol standard was obtained from Wako Pure Chemical Industries, Ltd. (Osaka, Japan). All other materials used in this study were of analytical grade.

### Preparation of administration samples

CSE was prepared by culturing *B*. *amyloliquefaciens* AS385 in 100 mL culture medium containing 4% Difco select soytone (BD Bioscience, Sparks, MD, USA) and 5% sorbitol at 37 °C with rotary shaking at 120 rpm. After 5 days of growth, 2,000 mL of culture was collected from twenty 500 mL Erlenmeyer flasks and centrifuged at 10,000 g for 5 minutes. Supernatant was collected, and citric acid crystal was added to adjust the pH to 3.0. The solution was centrifuged at 10,000 g for 5 minutes and filtered with glass filter (Whatman GF/A, Whatman, Maidstone, UK). Then, filtrate was applied to a cation exchange column (Amberlite IR-120B, Dow chemical, Midland, MI, USA, H^+^ form, 350 mL), followed by washing with water (1,750 mL), and elution with 1 M NH_3_. The eluate was fractionated into 200 mL portions and fraction with the highest DNJ concentration was added with dextrin and lyophilized. The amount of added dextrin was equal to the total solid contained in the eluate fraction. From this procedure, CSE containing 5% of DNJ and 50% of dextrin was obtained.

To prepare MLE, fresh mulberry leaves were blanched with steam at 90 °C for 90 seconds, then dried using hot air dryer at 40 °C for 40 minutes and powdered. 50 g of mulberry leaves powder was extracted with 1,000 mL of 50% methanol solution containing 0.1% acetic acid with constant shaking at 500 rpm for 24 hours at room temperature. Then, the extract was filtered through 5C filter paper and concentrated to 400 mL with rotary evaporator. The concentrate was acidified using formic acid until the pH reached 3.0, then applied to a cation exchange column (Amberlite IR-120, H^+^ form, 120 mL). The column was washed with water (1,000 mL), followed by elution of DNJ with 0.5 M NH_3_ (1,000 mL). The eluate was concentrated to 100 mL using rotary evaporator, mixed with equal amount of dextrin and lyophilized to obtain MLE, which is contained 5% DNJ and 50% dextrin.

For oral sucrose tolerance test, SEIBULE Tab. (50 mg miglitol/tablet) was obtained from Sanwa Kagaku Kenkyusho Co., Ltd. (Aichi, Japan) and DNJ was chromatographically isolated from culture medium of *B*. *amyloliquefaciens* according to our previous method [12].

### Anti-hyperglycemic effect of CSE

Oral sucrose tolerance test was carried out to evaluate the anti-hyperglycemic effect of CSE. Male Sprague-Dawley rats (7 weeks old; CLEA Japan, Tokyo, Japan) weighed 290–400 g were housed in a room with controlled temperature (23 ± 1 °C) and light (lights on from 08:00 to 20:00), had free access to distilled water and were fed commercial diet (CE-2; CLEA, Japan). At the end of one-week acclimatization period, the rats were fasted for 12 hours and divided into 5 groups (n = 6): control, CSE, MLE, miglitol, and DNJ group. CSE, MLE, miglitol and DNJ group received administration dose equivalent to 5 mg DNJ or miglitol/kg B.W. and control group received 1 mL of water. 15 minutes after sample administration, sucrose solution was orally administered (2 g/kg B.W.) to the rats. At 0, 15, 30, 45, 75, 105, 135, 195 and 255 minutes after sucrose solution administration, blood glucose level was measured from tail venous blood using glucose meter StatStrip Xpress 900 (Nova Biomedical, Tokyo, Japan), and blood was collected in heparinized capillary tube. Plasma was obtained by centrifugation at 1,000 g for 15 minutes at 4 °C. The aza-sugar composition in plasma was determined using LC-MS/MS as described in our previous reports [13]. Briefly, aza-sugars were extracted from plasma using mixture of water and acetonitrile, followed by sonication and centrifugation. Then, the aliquot was subjected to LC-MS/MS.

### Safety evaluation of microorganism DNJ

To evaluate the safety of microorganism DNJ, we measured the absorption and excretion of microorganism DNJ in rats using ^15^N-labeled DNJ. ^15^N-labeled DNJ was prepared using microorganisms followed by our previous study [12]. Male Sprague-Dawley rats (6 weeks old; CLEA Japan, Tokyo, Japan) were kept under the conditions as previously described. After one-week acclimatization period, rats were fasted for 12 hours, then received oral administration of ^15^N-labeled DNJ (10 mg/rat), while control group will receive oral administration of water. Urine and feces were collected using metabolic cages up to 48 hours after sample administration.

To determine the amount of ^15^N isotope in urine and feces, total amount of nitrogen atom and nitrogen isotope ratio (^14^N/^15^N) were measured using SUMIGRAPH NCH-22 (Sumika Chemical Analysis Service Ltd., Osaka, Japan) and ANCA-GSL analyzer (PDZ Europa Ltd., Sandbach, UK).

### Ethics

All animal studies were performed according to protocols approved by the Institutional Committee for Use and Care of Laboratory Animals of Tohoku University, which were granted by the Tohoku University Ethics Review Board (2014AgA-014 and 2016AgA-061).

### Statistical analysis

Data is expressed as means ± standard errors. All statistical analyses were performed using the excel statistical software package (BellCurve for Excel, Social Survey Research Information Co., Ltd., Tokyo, Japan). One-way analysis of variance (ANOVA) with Dunnett’s post hoc test was performed to compare the blood glucose concentration. Comparison of the plasma DNJ and miglitol concentration were carried out using One-way ANOVA with Tukey’s post hoc test. Differences between the means were significant at p < 0.05.

## Results and discussion

### Anti-hyperglycemic effect of CSE

Since the elevation of blood glucose (e.g. postprandial hyperglycemia) was involved in the development of vascular injury [14], it is important to control blood glucose to prevent and improve diabetes [15]. During the digestion, saccharides from foods are broken down by digestive enzymes and absorbed through the alimentary canal in forms of monosaccharides. Many studies have reported the effectivity of mulberry leaves in suppressing the elevation of blood glucose level [16]. Long-term administration of mulberry could also improve diabetes condition in rats [17]. It was revealed that DNJ content in mulberry leaves was responsible for these effects. For the past several decades, DNJ has been known as a potent α-glucosidase inhibitor. It prevents the absorption of carbohydrates in gastrointestinal tract by inhibiting the activity of enzymes that are responsible in the degradation of disaccharides to monosaccharides, resulting in the suppression of blood glucose elevation.

**Fig 2:**
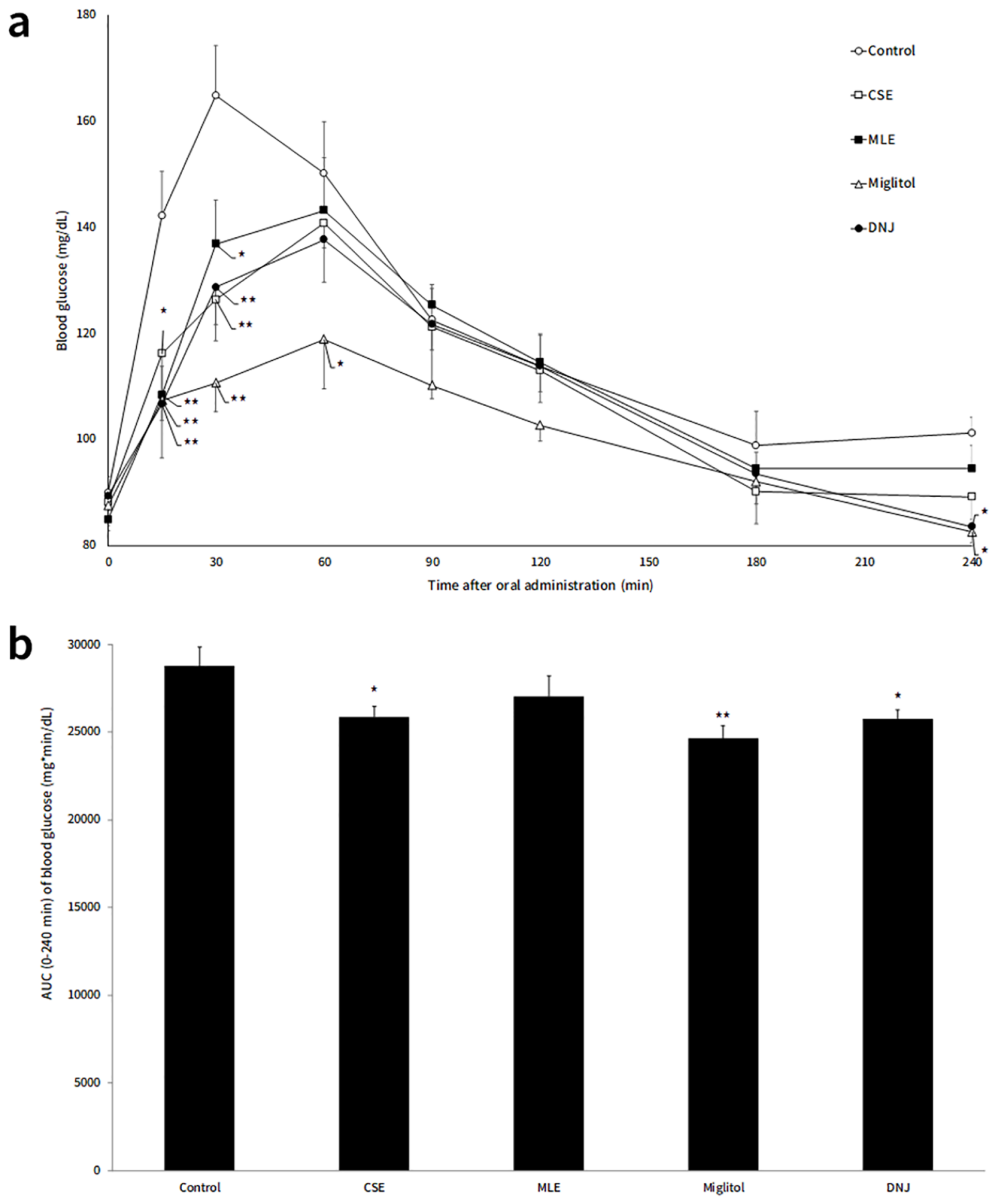
The effects in suppressing the blood glucose in rats. The rats received orally administered sucrose (2 g/kg B.W.) 15 minutes after each samples administration (equivalent to 5 mg DNJ or miglitol/kg B.W.). Blood glucose levels were determined from tail blood sample. (a) is the transition of blood glucose concentration and (b) is area of under curve of blood glucose concentration. Results are given as means ± SE. * p < 0.05 versus control; ** p < 0.01 versus control.

Therefore, it is expected that CSE containing DNJ could also contribute in prevention and improvement of diabetes as with MLE. To confirm this hypothesis, OSTT was performed to evaluate the anti-hyperglycemic effect of CSE. The result was shown in Fig 2. Compared with control group, the blood glucose level in CSE and MLE group was significantly decreased after 15 and 30 minutes of sucrose administration. There was no significant difference in the blood glucose-lowering effectivity between CSE and MLE groups, suggesting that CSE was able to prevent the onset and progression of diabetes just as effective as MLE.

**Fig 3:**
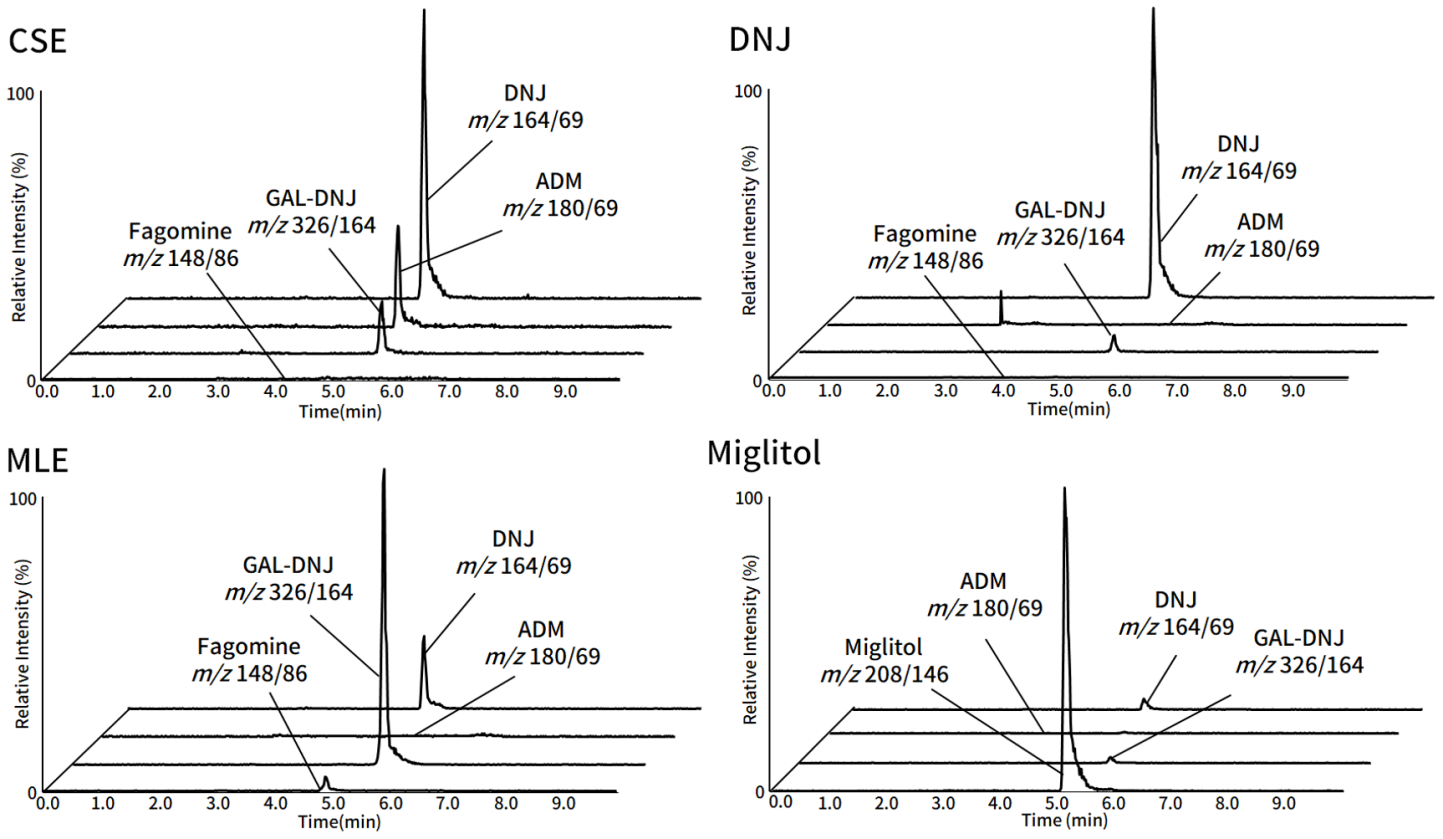
The chromatograms of administration samples. Aza-sugars in each sample were analyzed using LC-MS/MS.

**Fig 4:**
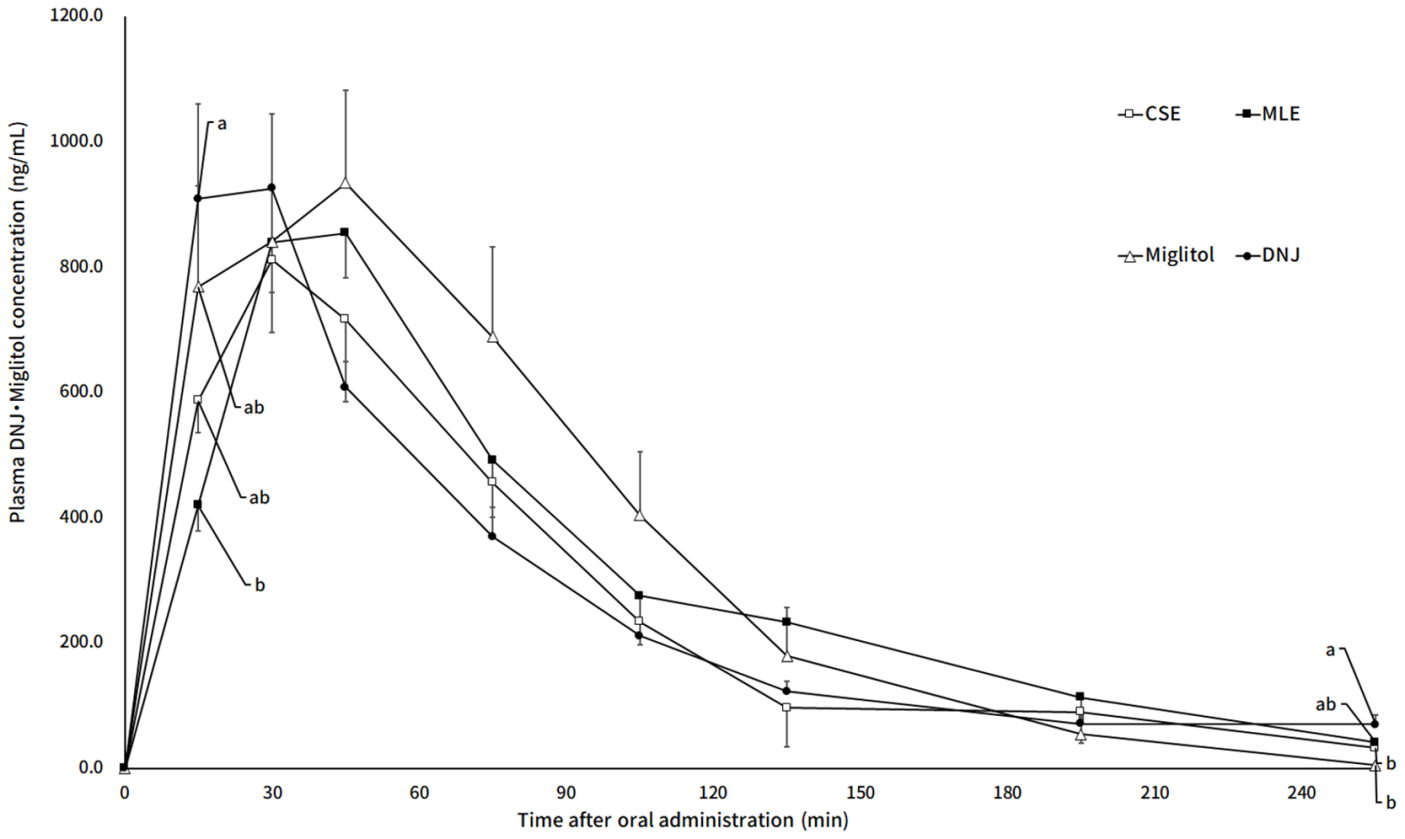
The transition of plasma DNJ concentration. The rats received orally administered sucrose (2 g/kg B.W.) 15 minutes after each samples administration (equivalent to 5 mg DNJ or miglitol/kg B.W.). Blood was collected from tail venous vein and plasma DNJ or miglitol concentration was determined using LC-MS/MS. Results are given as means ± SE. Means without a common letter differ significantly (p < 0.05).

In addition to DNJ, CSE and MLE contain other type of aza-sugars (e.g. 2-amino-2-deoxy-D-mannitol (ADM), 2-O-α-D-galactopyranosyl-deoxynojirimycin (GAL-DNJ), and fagomine) (Table 1 and Fig 3). Previous studies have reported the functional properties of these aza-sugars. Fagomine, which has similar structure with DNJ have shown α-glucosidase inhibitory activity [18] that may be linked to induced insulin release [19]. GAL-DNJ exhibited anti-hyperglycemic activity in rat [20]. Considering these findings, the effect of these aza-sugars in administration samples on the suppression of blood glucose level was originally taken into account. To confirm this, we compared the effect of MLE and CSE with DNJ. The result demonstrates similar trend on blood glucose level among these three groups (CSE, MLE and DNJ group), which suggested that the other aza-sugars (ADM, fagomine and GAL-DNJ) have no impact on the anti-hyperglycemic activity. Even if considering the limited content of ADM and fagomine in CSE and MLE, it was suggested the role of DNJ as the main anti-hyperglycemic compound in CSE. Moreover, the plasma concentration of DNJ after oral administration of CSE, MLE or DNJ in rats (Fig 4) was relatively similar, indicating that the behavior of DNJ in the small intestine was similar among three group (CSE, MLE and DNJ group). This could explain the similarity in blood glucose-lowering effect that occurred among these groups. Moreover, CSE and MLE were also contained 50% of dextrin. While we were unable to accurately determine the composition of the rest 45% portion of the CSE and MLE, we assumed that some amino acid, sugar containing nitrogen and so on were contained in CSE and MLE, suggested not affected to the anti-hyperglycemic effect.

**Table 1:** The aza-sugars composition of administration samples.

Administration samples	Composition (% dry weight)				
	DNJ	GAL-DNJ	Fagomine	ADM	Miglitol
CSE	4.7	0.1	N.D.	1.4	N.D.
MLE	5.0	4.6	0.5	N.D.	N.D.
DNJ	89.4	trace	N.D.	trace	N.D.
Miglitol	trace	trace	N.D.	N.D.	24.7
					Trace < 0.1%

Each sample was analyzed using LC-MS/MS, and the composition was calculated from calibration curve using each standard material.

The present study showed that the suppressive effect of miglitol was higher than that of DNJ, which was consistent with the previous findings. Although the anti-hyperglycemic effect of miglitol and DNJ have hardly been compared in vivo, miglitol showed stronger inhibition towards rat sucrase when compared to DNJ in vitro [21]. The suppressive effect of alpha glucosidase inhibitor, such as DNJ and acarbose is dependent on the administration dose [1,22], thus DNJ can be used for prevention of diabetes like a drug miglitol.

### Absorption and excretion of microorganism DNJ

In this study, we confirmed the effectivity of CSE in suppressing the blood glucose level and identified DNJ as the active component in CSE. Moreover, CSE exhibited α-glucosidase inhibitory activity in small intestine, and presence of DNJ deriving from CSE was also detected in the blood. While these findings suggested the potential use of microorganism DNJ for therapeutic purposes, detailed information regarding its safety were still limited. For this reason, we verified the safety of microorganism DNJ by evaluating its absorption and excretion after oral administration in rats.

First, to evaluate the absorption and excretion of DNJ, we prepared and administered ^15^N-labeled DNJ to rats, then collected the urine and feces. The amount of ^15^N from DNJ in urine and feces was determined by measuring the total amount of nitrogen and isotope ration of nitrogen (^14^N/^15^N). Few studies have reported the absorption and excretion of DNJ. Yang et al. reported the absorption and rapid excretion of DNJ, fagomine and 1,4-dideoxy-1,4-imino-D-arabinitol in rats. [23]. Amezqueta et al. reported partial absorption and metabolism of DNJ and fagomine. [24]. Both studies utilized LC-MS/MS apparatus for DNJ quantification. Although the advantages of LC-MS/MS are high selectivity and sensitivity, it is difficult to detect and quantify all metabolites. In this study, we have succeeded to quantify DNJ and its metabolite all at once in urine and feces using ^15^N-labeled DNJ.

**Fig 5:**
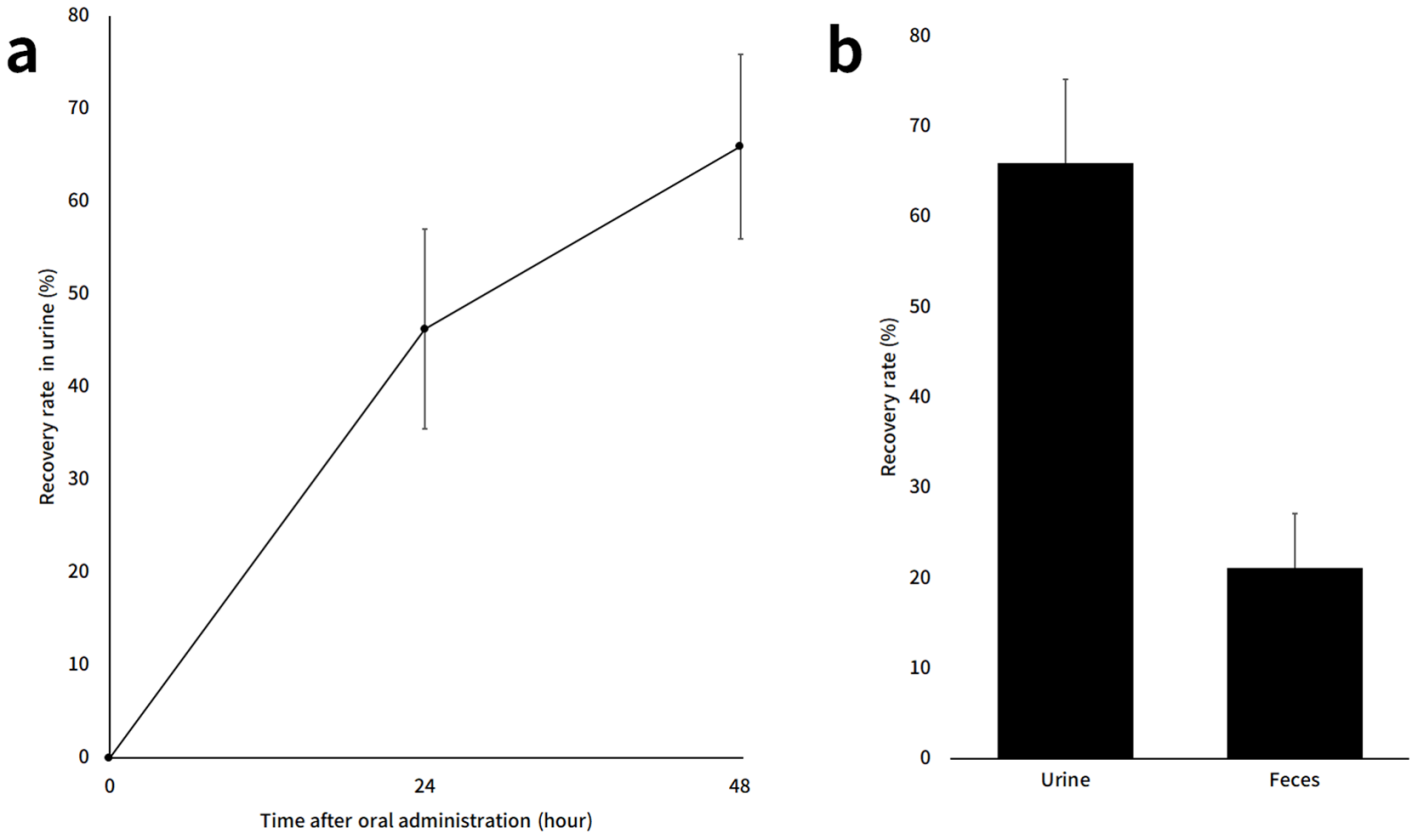
The recovery rate of ^15^N from DNJ in urine and feces until 48 hours after sample administration. The recovery rate of ^15^N from DNJ in urine and feces until 48 hours after sample administration. After 12 hours fasting, the rats were received ^15^N-labeled DNJ (10 mg). Urine and feces were collected and the amount of ^15^N from DNJ was analyzed. (a) The time course of recovery rate of ^15^N in urine. (b) Total recovery rate of ^15^N in urine and feces. Results are given as means ± SE.

The recovery rate of ^15^N from DNJ in urine and feces was shown in Fig 5. The isotope ratio of control group was similar with that of atmospheric air (data not shown). Although the values varied among individuals, approximately 50% or more DNJ-derived ^15^N was obtained from urine, suggesting that over half of the DNJ administration dose was absorbed after administration of 10 mg ^15^N-labeled DNJ. We also found that the recovery rate of ^15^N from DNJ reached 80% after 48 hours of DNJ administration, corresponding with Amezqueta’s findings. This finding suggested that DNJ is hardly metabolized and rapidly excreted. The pharmacokinetics of miglitol, N-hydroxyethyl derivative of DNJ was studied using the radio isotope labeling method [25]. After oral administration, miglitol was excreted from the body within 48 hours, just like DNJ in this study. Miglitol has been utilized as anti-diabetes drug and appears to be safe for human consumption with mild side effects including abdominal distension and diarrhea [26]. The similarities in absorption and excretion pattern of miglitol and DNJ, therefore, suggested the safety of DNJ.

While 80% of ^15^N from DNJ was recovered, the fate of the remaining 20% of ^15^N was still unclear. It is highly possible that DNJ was distributed the organs and tissues. In the previous studies, the presence of DNJ in organs was detected after single administration [23] and long-term administration [27]. Distribution of DNJ may contribute to its beneficial effects in the body and be concerned any side effects. While this study targeted the evaluation of anti-hyperglycemic effect of microorganism DNJ which exhibited in small intestine epithelium, DNJ have also been reported the targeted therapeutic effects including the improvement of lipid metabolism [28], anti-viral [29] and anti-cancer [30] activity. Moreover, it was reported the improvement of obesity through reduction of hypothalamic endoplasmic reticulum stress when intracerebroventricular administration [31], implying the effectiveness of DNJ in the body. Therefore, to understand the underlying mechanism that responsible for DNJ therapeutic effects, further study needs to be done to get more insight into the distribution of DNJ and its metabolites in organs, expected the contribution of ^15^N labeling method for its elucidation. While the distribution of DNJ into organs and tissues may be responsible for its therapeutic effect, the possible adverse effect that resulted from disposition of DNJ in organs has also become a major concern, to address this issue, we are planning to carry out a more thorough safety evaluation on DNJ, referring to established guideline such as Safety Pharmacology Studies for Human Pharmaceuticals [32].

## Conclusion

We confirmed the effectivity of microorganism DNJ (CSE) in suppressing the elevation of blood glucose level and identified DNJ as the main active component in CSE. The blood glucose-lowering effect of CSE was comparable with that of MLE, suggesting its potential role in prevention and treatment of diabetes. To confirm the safety of microorganism DNJ, we succeeded to develop the quantification method targeting DNJ and its metabolite using ^15^N-labeled DNJ, and the absorption and excretion of microorganism DNJ were evaluated. We found that most of the administered DNJ was absorbed and rapidly excreted from the body up to 48 hours after oral administration, just like anti-diabetic drug miglitol, suggesting the safety of DNJ. On the other hand, small parts of administered DNJ was possibly distributed to organs or tissues, expected further study to clarify the distribution of DNJ using ^15^N labeling method. Overall, we confirmed the functional properties and safety of microorganism DNJ and therefore suggesting its utilization for therapeutic purposes.
